# The effect of heat-sensitive moxibustion in the elderly with nocturia: protocol for a randomized controlled trial

**DOI:** 10.3389/fmed.2025.1508731

**Published:** 2025-08-12

**Authors:** Jinhua Geng, Xintong Ma, Yihui Lin, Qiaoli Lin, Kaitian Wen, Chunyan Ruan, Zunjiang Li, Zhaofan Mo, Shuang Li, Banghan Ding, Na Liu

**Affiliations:** ^1^Emergency Department, Guangdong Province Hospital of Chinese Medicine, Guangzhou, China; ^2^The Second Affiliated Hospital of Guangzhou University of Chinese Medicine, Guangzhou, China; ^3^The Second Clinical College of Guangzhou University of Chinese Medicine, Guangzhou, China

**Keywords:** nocturia, elderly, heat-sensitive moxibustion, traditional Chinese medicine, clinical trial design

## Abstract

**Background:**

Nocturia is a common and bothersome symptom in the elderly population, significantly impacting their quality of sleep and overall well-being. Heat-Sensitive moxibustion (HSM) is effective for elderly people with nocturia, but its efficacy has not been scientifically proven. This randomized controlled trial is designed to determine the efficacy and safety of HSM in the elderly with Nocturia.

**Method:**

This study is designed as a randomized, single-blind clinical trial, focusing on a population of 110 patients aged 60 and above who suffer from non-specific nocturia lasting for at least 1 month. Patients in the intervention arm are scheduled for 2 cycles of HSM, while patients in the control group will undergo the same cycle of conventional treatment. To ensure objectivity, assessors and investigators will remain blinded to the randomization process. The primary outcome of this study is the reduction in the frequency of nocturia. Additionally, several secondary outcomes will be evaluated, including sleep quality, quality of life scores, the efficacy of traditional Chinese medicine (TCM) syndrome, and changes in blood biomarkers Arginine vasopressin (AVP) and Atrial natriuretic peptide (ANP). These assessments will be conducted at baseline, post-intervention and at the 2-month follow-up to comprehensively evaluate the effectiveness and safety of HSM.

**Discussion:**

In this study, we aim to quantitatively analyse the therapeutic effect and safety of HSM on nocturia in the elderly, clarify the amount of moxibustion, the distribution pattern of heat-sensitized acupoints, the frequency of heat-sensitized acupoints, and the moxibustion sensation for the treatment of senile nocturia, which can bring insights into future clinical treatments.

## 1 Introduction

Nocturia is a condition where an individual experiences the necessity to awaken at least twice during the night for the purpose of urination, which is associated with a multitude of adverse health sequelae, including the exacerbation of sleep disturbances, the induction of depressive states, and a concomitant deterioration in the quality of life (1). The prevalence of nocturia was ranging from 11% to 44% among populations aged 20–40 years, while 28%–62% of the elderly (> 70 years old) voiding two or more times per night (2). Recent studies have shown that as the frequency of nocturia increases, mortality increases in a frequency-dependent manner, which may be related to impairment of physiological nocturnal blood pressure drop, increased sympathetic nerve activity, and insulin resistance (3, 4). However, this symptom has not garnered the attention it deserves until now. It was reported that 50 million people are affected by this disorder in the US, but only 1.5 million receive treatment (5). The pathophysiology of nocturia is complex and multifactorial, and may originate from the urological system (lower tract symptoms) or non-urological system, including chronic heart failure, hypertension, diabetes, sleep disorders, etc. Nocturia is characterized by disrupted circadian regulation of fluid homeostasis, and two key hormonal pathways were prioritized based on their mechanistic relevance. Current research has found that arginine vasopressin (AVP) and the water homeostasis biological system are closely related to the occurrence of nocturia. AVP is the primary hormone regulating water homeostasis, promoting renal water retention through increased osmotic driving force and transcellular water transport in epithelial cells (mediated by the cGMP-PKG signaling pathway and AQP2-binding proteins), ultimately enhancing urine concentration (6). Consequently, a deficiency in AVP leads to decreased blood osmolality and subsequent increase in water excretion, resulting in clinical manifestations such as polyuria and nocturia. Another class of hormones involved in water salt balance, the diuretic peptide family, may also play a role. Atrial natriuretic peptide is secreted by the right atrium under the stimulation of atrial wall expansion and is sensed by atrial volume receptors, accounting for the majority of the total amount of diuretic peptides in the circulation (7). A multicenter retrospective cohort study in Japan showed that patients with nocturia had elevated ANP levels, and even higher ANP levels in urine (8). The management strategy of nocturia encompasses a multidisciplinary approach, including pharmacological interventions, notably desmopressin, diuretics, antimuscarinics, and non-steroidal anti-inflammatory drugs (NSAIDs), physical therapy and regular follow-ups (9). In patients receiving desmopressin for the treatment of nocturia, the incidence of hyponatremia is 4.4%, influenced by factors such as age, comorbidities, concurrent medications, and reduced estimated glomerular filtration rate (eGFR) levels (10). Clinicians should implement routine monitoring protocols, including serum sodium checks, particularly in high-risk populations to ensure the safe and effective use of desmopressin (11). While these interventions exhibit certain therapeutic benefits, their limitations such as poor specificity, pronounced side effects, protracted treatment durations, and substantial costs often yield outcomes that fall short of expectations (12). Hence, the development of novel and alternative medical management strategies for nocturia remains a challenging task and is urgently required.

Heat-sensitive moxibustion is a moxibustion therapy focused on the “heat sensitivity” of acupoints and the qi-obtaining effect of moxibustion (13), with the advantage of featuring simplicity, safety, effectiveness, and non-toxic side effects (14). When performing moxibustion over traditional meridians and acupoints, if a distinct area experiences one or more phenomena of heat penetration, heat transfer, or thermal conductivity, that specific location can be precisely identified as the heat-sensitive acupoint. In contrast to mild moxibustion, applying specific techniques to stimulate the qi-obtaining effect of moxibustion and achieving individualized desensitization moxibustion at heat-sensitive acupoints can significantly improve the therapeutic effect (15). A randomized controlled trial demonstrated that HSM achieved a total effective rate of 92.0% in migraine treatment, significantly superior to the 72.0% efficacy observed with mild moxibustion (16). Researchers have verified that the thresholds pertaining to heat perception, heat-induced pain, and heat pain tolerance are markedly elevated at heat-sensitive acupoints in comparison to non-heat-sensitive counterparts by using quantitative temperature sensation assessment techniques, thereby furnishing robust support for the genuine presence of the heat-sensitive state within acupoints (17). Over the past years, HSM has gained extensive application in treating a range of conditions affecting the cardiovascular, digestive, nervous, sports systems and so on (18). Meanwhile, limited trials had revealed that HSM can significantly reduce the frequency of nocturia, the degree of urinary frequency, and improve the quality of life and sleep in patients with nocturia, making it an effective treatment for nocturia that is worthy of clinical promotion and application (19). The mechanism of HSM in treating diseases exhibits a multi-target, multi-dimensional, and multi-pathway approach, primarily involving inflammatory response, immune regulation, cell proliferation and apoptosis, as well as hormonal balance within the body (20). The integrative effect of multiple brain regions showing significant changes aligns with the somatic pain-temperature conduction pathway following HSM, suggesting that somatosensory system activation may serve as the neurobiological foundation for meridian sensation transmission, while the coordinated network of these regions mediates HSM’s therapeutic effects through ACC-IC medial pain system modulation of emotional network integration (21). Mechanistically, HMS may normalize circadian hormone secretion through central neural activity. Despite the promising potential of HSM for the elderly with nocturia, there is a notable paucity of clinical randomized controlled trials (RCTs) with high qualified evidence in the literature to date.

Thus, further extensive and rigorous RCTs are warranted to comprehensively assess the safety, efficacy, and applicability of this therapeutic approach in elderly patients, which not only could advance our understanding of the mechanism and outcomes of heat-sensitive moxibustion but also provide the necessary scientific evidence to support its clinical application for the management of senile nocturia.

## 2 Methods

### 2.1 Trial design

**FIGURE 1:**
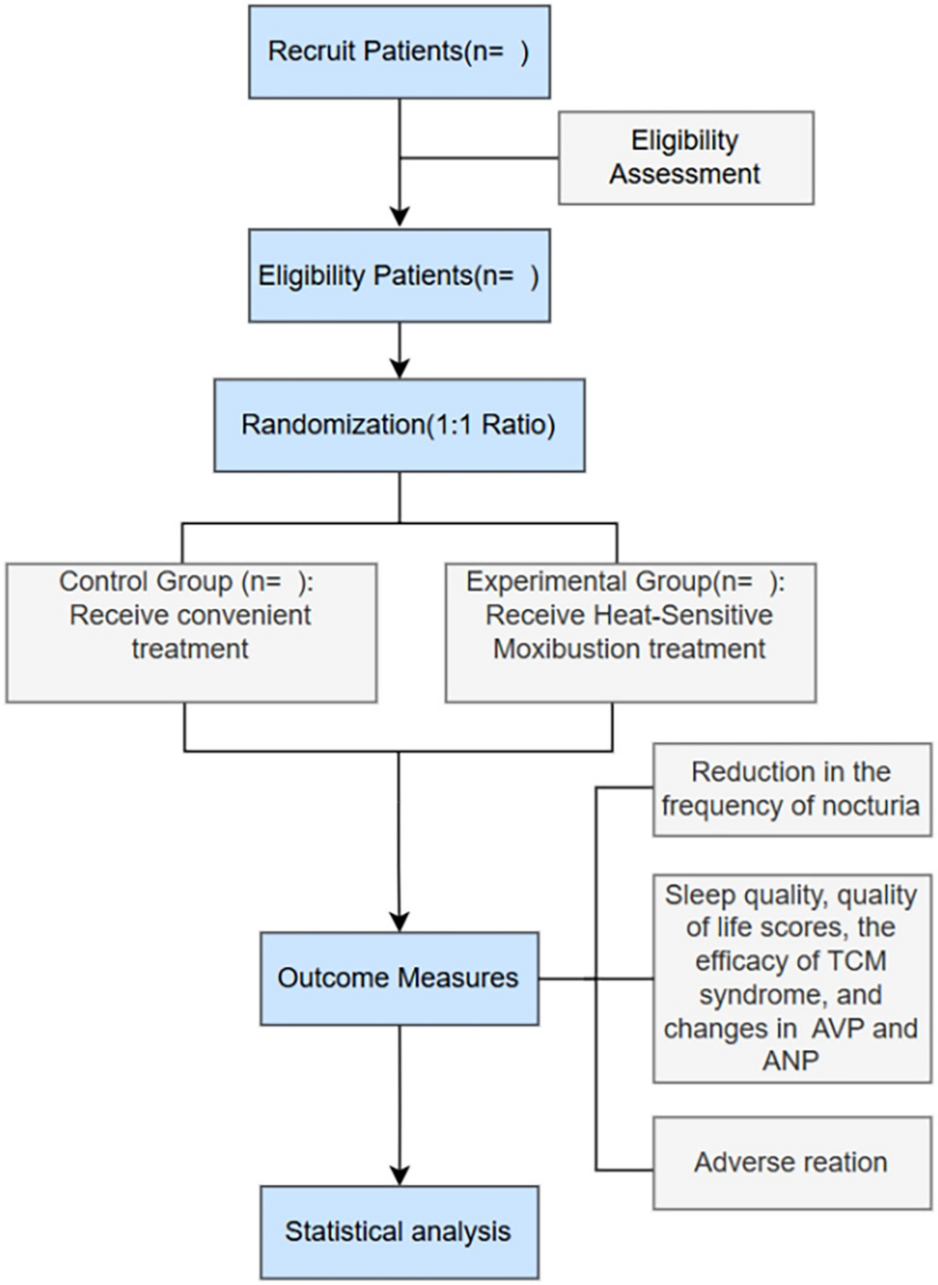
The flow chart of the clinical procedures.

The trial utilizes a prospective, randomized and single-blind design. A flow diagram of the study design is shown in Figure 1, and the study schedule is depicted in Table 1.

**TABLE 1:** Schedule of enrollment, intervention and assessment.

Time point	Enrollment Week -1	Allocation Week 0	Treatment phase Week 1-2	Follow-up Week 3	Follow-up Week 10
**Assessment of eligibility**					
Eligible screen	X	–	–	–	–
Informed consent	X	–	–	–	–
**Randomization**					
Allocation	–	X	–	–	–
**Intervention**					
Conventional treatment	–	–	X	–	–
Heat-sensitive moxibustion plus conventional treatment	–	–	X	–	–
**Primary outcome**					
The frequency of nocturia	–	X	–	X	X
**Secondary outcomes**					
Pittsburgh sleepiness index (PSQI) scale	–	X	–	X	X
Nocturia on quality of life (N-QOL) questionnaire	–	X	–	X	X
Traditional Chinese medicine syndrome scoring scale	–	X	–	X	X
The level of arginine vasopressin (AVP)	–	X	–	X	X
The level of atrial natriuretic peptide (ANP)	–	X	–	X	X

### 2.2 Study setting

The project department is the Comprehensive Third Department at Guangdong Provincial Hospital of Traditional Chinese Medicine, which has a wide range of case sources, ensuring the availability of case data for this project. This prospective clinical trial will be conducted between January 1, 2024 and December 31, 2025.

### 2.3 Research hypothesis

Primary hypothesis: HSM groups will experience significant improvement in the frequency of nocturia.

Secondary hypothesis: HSM groups are anticipated to exhibit notable enhancements in sleep quality, quality of life, as well as traditional Chinese medicine (TCM) syndrome scores.

### 2.4 Study participants

#### 2.4.1 Inclusion criteria

① Complied with the Western medical diagnostic criteria “an individual wakes up one or more times during the night to void with each void preceded and followed by sleep or the intention to sleep,” combined with at least one diagnosed pathology, including benign prostatic hyperplasia and overactive bladder (22) and complied with the traditional Chinese medicine kidney yang deficiency syndrome diagnostic criteria for nocturia (The second edition of Differential Diagnosis of Traditional Chinese Medicine Syndromes); ② Patients aged ≥ 60 years, regardless of gender; ③ The patient’s duration of illness is 1 month at least; ④ Normal urine routine test results and negative urine bacterial culture; ⑤ Patients with a sense of urination and the ability to control urination; ⑥ Patients with clear consciousness, rational mentality, cooperative behavior, articulate speech, who could accurately express their own wishes and communicate their moxibustion experience correctly with the practitioner; ⑦ Patients who are willing to participate in the study and have signed the informed consent form.

#### 2.4.2 Exclusion criteria

① Individuals who experience dizziness during moxibustion or have allergies or intolerance to mugwort smoke; ② Patients with large areas of skin lesions, ulcers, or sensory impairment at the moxibustion site; ③ Patients with urinary tract infections evidenced by positive urine routine tests or urine bacterial cultures, accompanied by or without symptoms such as urgency and pain during urination; ④ Patients diagnosed with stress incontinence, urge incontinence, mixed incontinence, overflow incontinence, or individuals requiring indwelling urinary catheterization; ⑤ Patients with urinary system organic diseases such as nephritis, renal cell carcinoma, ureteral stones, and bladder cancer; ⑥ Patients with severe primary diseases in the cardiovascular, cerebrovascular, liver, kidney, hematopoietic, or endocrine systems, as well as those with intellectual disabilities; ⑦ Patients who have undergone surgery, have tumors, or have unstable blood pressure or blood sugar levels; ⑧ Critically ill patients, those with severe mental illnesses, or those with communication barriers such as speech difficulties; ⑨ Patients who have taken medications that could interfere with this study within the last week, such as diuretics, or are currently receiving medication or non-pharmacological treatments for nocturia; ⑩ Patients who are currently participating in clinical trials for other medications or treatments and withdraw from the study voluntarily or are lost to follow-up.

### 2.5 Intervention

#### 2.5.1 Control group: conventional treatment

Based on the Chinese Expert Consensus on Clinical Diagnosis and Treatment of Nocturia published in the Chinese Journal of Urology in 2018, a health education program is designed for research subjects who meet the inclusion criteria, focusing on daily lifestyle habits and exercise routines. The program requires daily implementation by the research subjects.

(1) Restrict fluid intake before bedtime, especially alcohol and coffee and empty the bladder as much as possible before going to bed to improve sleep quality.

(2) Maintain warmth during the night to increase skin blood supply and reduce urine production.

(3) Engage in moderate exercise and elevate the lower limbs to reduce water retention.

(4) For patients with Overactive Bladder (OAB), perform bladder function exercises such as delayed urination.

(5) Conduct pelvic floor muscle exercises.

#### 2.5.2 Experimental group: heat-sensitive moxibustion plus conventional treatment

(1) Acupoint Selection: Qihai, Guanyuan, Ciliao (bilateral), Yongquan (bilateral).

(2) Acupoint Location: Refer to the national standard of the People’s Republic of China, “Names and Locations of Acupoints” (GB/T123456-2006).

(3) Usage: After urination, the patient should adopt a comfortable posture, exposing the skin at the designated acupoints while ensuring sufficient warmth. The practitioner will then embark on a suspended moxibustion exploration, utilizing a blend of tracing, rotating, pecking, and gentle moxibustion techniques within specific regions: the abdomen (centered on the Ren Meridian, spanning from Qihai to Guanyuan, with a 3 cm radius), the lower back (focusing on both Ciliao acupoints, also with a 3 cm radius), and the soles of the feet (centering on both Yongquan acupoints, maintaining a 3 cm radius). Subsequently, a tracing moxibustion will be administered, where a lit pure moxa stick is held approximately 3 cm away from the patient’s skin and smoothly traversed along the Ren Meridian, Bladder Meridian, and Kidney Meridian. This process continues until the patient experiences a warm sensation along the treatment path, facilitating the clearing and activation of the meridians. This tracing moxibustion phase lasts for 2–3 min. Following this, a rotating moxibustion will be applied to the acupoints for 1–3 min, aiming to warm and stimulate local qi and blood circulation while identifying sensitive points. These sensitive points manifest as various heat-sensitive sensations, including heat penetration, heat expansion, or heat conduction along meridians, among others. Any acupoint that elicits one or more of the nine types of heat-sensitive moxibustion sensations (e.g., warmth in extremities, body warmth, preference for heat, skin erythema, facial flushing or sweating on the forehead, gastrointestinal motility reactions, heat penetration, heat expansion, or non-thermal sensations) is deemed a heat-sensitive acupoint, regardless of its alignment with traditional acupoint locations. The selection of optimal heat-sensitive acupoints follows a specific hierarchy: acupoints with non-thermal sensations are prioritized; those where the heat-sensitive sensations direct or reach the affected area are preferred; and acupoints exhibiting stronger heat-sensitive sensations are given higher priority. Next, a pecking moxibustion will be applied for 1–2 min to intensify the heat sensitivity of the chosen acupoints. This is followed by a gentle moxibustion technique to initiate and propagate the heat-sensitive sensations until they dissipate or significantly diminish.

One treatment cycle consists of 5 days of treatment followed by 2 days of rest, with 2 consecutive treatment cycles being administered. To isolate the effects of the experimental intervention, all enrolled participants (both intervention and control groups) will be instructed to refrain from receiving any additional moxibustion treatments or traditional Chinese medicine therapies (e.g., acupuncture, cupping, herbal medicine) during the 8-week study period. These restrictions were communicated verbally and in written form at enrollment. And participants will be requested to complete weekly diaries to report any inadvertent use of prohibited therapies.

### 2.6 Treatment of adverse reactions

If blisters or burns arise locally due to excessive moxibustion or accidental mishandling, promptly administer a specialized burn ointment. For minor blisters, no specific treatment is necessary. However, for larger blisters, fluid extraction is imperative. Following fluid extraction, ensure the affected areas are covered with sterile gauze to protect them from water and prevent infection. In cases of severe conditions, seek medical attention promptly.

### 2.7 Procedure

Prior to the commencement of therapy, the practitioner ought to document the patient’s nocturia frequency, administer and record the Pittsburgh Sleep Quality Index (PSQI) and Nocturia Quality of Life (N-QOL) scale scores, assess the traditional Chinese medicine syndrome score, and evaluate the intensity of nocturia and urinary frequency. Upon the morning subsequent to enrollment, fasting blood samples will be procured for the assessment of AVP and ANP levels. Throughout the treatment period, nocturia frequency will be meticulously recorded on a daily basis.

Upon completion of the treatment regimen, the PSQI, N-QOL scale scores, and traditional Chinese medicine syndrome score will be reassessed, and fasting blood samples will be collected the subsequent morning for a repeat analysis of AVP and ANP. Additionally, 2 months post-treatment, these assessments–including the PSQI, N-QOL scale scores, traditional Chinese medicine syndrome score, and fasting blood tests for AVP and ANP–will be conducted once more.

Ultimately, a comprehensive evaluation will encompass the nocturia frequency, sleep quality, quality of life, traditional Chinese medicine syndrome scores, as well as AVP and ANP levels, for both patient cohorts, both pre- and post-treatment, with a further assessment conducted 2 months after the conclusion of therapy. Figure 1 illustrates the clinical procedures during the research.

### 2.8 Outcome measures

#### 2.8.1 Primary outcomes

The frequency of nocturia: 72-hour urination diary.

#### 2.8.2 Secondary outcomes

(1) Sleep quality: Pittsburgh Sleepiness Index (PSQI) and sleep duration. The PSQI scale is used to assess the sleep quality of the patient in the past month. It consists of 5 other-rated items and 19 self-rated items. All other-rated items and the 19th self-rated item are not scored. The 18 items involved in the scoring are divided into 7 parts: sleep quality, sleep onset, actual sleep time, sleep efficiency, sleep interference, drug-assisted sleep, and daytime energy. Each part is scored 0–3 points according to the level, and the scores of each item are added up to the total score of the PSQI scale. The score range is 0–21 points. The higher the score, the more severe the insomnia; conversely, the lower the score, the better the sleep quality.

(2) The quality of life: Questionnaire on the impact of nocturia on quality of life (N-QOL scale score). The N-QOL scale is used to assess the quality of life of the test subject in the past 2 weeks. It is divided into 3 dimensions and 13 questions, including 7 questions on energy/sleep (0–28 points), 5 questions on distress/concern (0–20 points), and 1 question on self-assessment of quality of life (0–4 points). Each question is scored 0–4 points. Questions 12 and 13 are reverse-scored questions with a score range of 0–52 points. The lower the score, the lower the quality of life, and vice versa.

(3) Therapeutic efficacy of traditional Chinese medicine syndromes: TCM syndrome scoring scale. Record the patient’s TCM symptoms, including tongue and pulse, nocturia, dry mouth and drinking water, etc. The higher the score, the more serious the patient’s condition.

(4) The level of AVP and ANP.

#### 2.8.3 Safety

All adverse events and vital signs will be observed and reported according to the standard operating protocol (SOP), especially the integrity of the skin at the moxibustion site.

### 2.9 Sample size

Based on relevant research (significance level, take α = 0.05, β = 0.10, test power 1-β = 0.90; two-sided test, confidence level 95%, *Z* = 1.96, *E* = 10%, *P* = 0.5), the estimated sample size is 96 cases preliminary. If the dropout rate is considered to be at most 15%, the total number of cases in the two groups should be at least 110. Therefore, 55 participants were recruited in each group.

### 2.10 Randomization and allocation concealment

A third party other than the researchers and evaluators uses a software package to generate random numbers and groups, and compiles and keeps random allocation cards, which are placed in sealed opaque envelopes in sequence. Qualified subjects open the random envelopes with corresponding numbers in the order of inclusion in the trial. In this way, the subjects are randomly divided into the treatment group and the control group, and the operation is carried out strictly according to the grouping in the envelopes.

### 2.11 Assignment of interventions: blinding

Blinding of participants was not feasible in this trial due to the inherent sensory characteristics of moxibustion (e.g., visible smoke, distinct thermal sensation, and herb aroma). To mitigate potential bias arising from participants’ awareness of treatment allocation, outcome assessors, data entry personnel, and statisticians will be blinded to group allocation throughout the study. In addition to patient-reported outcomes, we incorporated objective biomarkers (e.g., ANP and AVP) as secondary endpoints to reduce reliance on subjective assessments. Meanwhile, practitioners delivered a neutral script to all participants before treatment, “This study aims to compare different approaches to managing your condition. Some treatments may produce physical sensations, but we ask you to focus on reporting your symptoms as accurately as possible, regardless of your expectations.”

### 2.12 Data collection and analysis

The data of various observation indicators of patients were collected on the day of treatment, the day after the end of treatment and 2 months after the end of treatment. To ensure the accuracy and completeness of the data, we will adopt a double-entry method. PASW Statistics 18.0 software was used to establish a database for case investigation data that met the inclusion criteria, and data review was performed after data entry was completed.

The statistical analysis of the PASW Statistics 18.0 data was performed, encompassing descriptive statistics for clinical baseline data. For continuous outcomes, group differences will be analyzed using Student’s *t*-tests if data are normally distributed; otherwise, the Mann-Whitney U test will be applied. For categorical variables, Chi-square tests or Fisher’s exact tests will be used as appropriate. Efficacy evaluations utilized both intention-to-treat (ITT) and per-protocol (PP) populations, while safety assessments were conducted on the safety analysis set. In the intention-to-treat analysis, missing data due to withdrawal or dropout will be handled using the last observation carried forward (LOCF) method. Participants who received prohibited therapies were retained in the trial per the ITT principle but flagged for sensitivity analysis. Their data were analyzed both within the full cohort and in a subgroup excluding protocol violators. When necessary, multiple imputation methods will be considered for missing value replacement, or alternative approaches will be selected based on actual circumstances. For clustering, the K-MEANS clustering method was utilized, while applying Pearson or Spearman correlation analysis to examine correlations. A *P*-value of 0.05 or less was considered statistically significant.

### 2.13 Quality oversight

To ensure protocol adherence and data integrity, a comprehensive quality oversight system was implemented:

(1) Monthly progress reviews will be conducted by the study management team to evaluate protocol adherence, recruitment progress, and data quality metrics; (2) An independent Data Monitoring Committee (DMC) comprising external statistician, clinician, and ethicist with no conflicts of interest will conduct biannual safety reviews and assess pre-defined stopping criteria; (3) Participant compliance will be tracked through returned moxa stick counts and treatment attendance, with deviations (> 20% missed sessions) escalated to the DMC; (4) Ethical oversight included institutional review board audits of adverse event reporting. All data modifications required dual approval (PI and DMC chair) post-audit, aligning with International Council for Harmonization-Good Clinical Practice Guideline (ICH-GCP) monitoring standards (23).

## 3 Discussion

Nocturia is a common age-related disease caused by multiple factors, rooted in circadian dysregulation of fluid homeostasis, which seriously affects the physical and mental health and quality of life in the elderly (24). HSM uses the medicinal power and heat of moxa fire to give the human body warm stimulation, stimulate human energy, and regulate the yin and yang balance of the internal organs through the conduction of meridian acupoints, ultimately achieving the purpose of harmonizing qi and blood and enhancing the body’s own resistance and defense. And further research indicated that the occurrence of heat-sensitive phenomenon is related to the plasticity changes of the brain neural network and the involvement of central glutamatergic neurons (25). While aging-related decline in AVP secretion is a well-established contributor, emerging evidence implicates ANP as a key mediator of nocturnal polyuria in non-cardiac populations. HSM may address this dual hormonal imbalance through its unique neuroendocrine modulation. The efficacy of HSM in preventing and treating diseases is much better than that of traditional moxibustion therapy. Compared with traditional moxibustion, HSM emphasizes the thermal moxibustion sensation and meridian qi transmission generated during the moxibustion process. By finding the thermal points for moxibustion to stimulate the meridian qi transmission, a small stimulus and a large response are produced to improve the efficacy (26). From a mechanism perspective, HSM may enhances therapeutic precision through three synergistic innovations. First, heat-sensitive points optimizes acupoint selection by dynamically targeting acupoint with aberrant thermal reactivity, a RCT focus on chronic fatigue syndrome showing that the total effective rate of the HSM group was 19.36% higher than that of the mild moxibustion group (93.55% vs 74.19%, *P* < 0.05) (27). Second, dose optimization is achieved by titrating moxa duration to individual “moxibustion sensation” thresholds, from warmth to radiation, and then propagation, ensuring adequate neurostimulation while avoiding thermal injury. Third, central plasticity modulation underpins HSM’s sustained effects: fMRI evidence reveals enhanced functional connectivity within the default mode network (DMN), particularly between the posterior cingulate cortex and hypothalamus (28), suggesting HSM may reset circadian rhythm centers by rewiring neural circuits governing AVP/ANP secretion. Together, these advances position HSM as a paradigm shift in precision moxibustion, bridging empirical practice with neurobiological validation. Recent studies have shown that HSM is more effective than mild moxibustion in reducing the frequency of nocturia, improving the degree of frequent urination, improving sleep quality and quality of life, and reducing the amount of residual urine in the bladder (28). However, there is a scarcity of clinical observational studies on the treatment of nocturia utilizing HSM, resulting in an insufficient amount of clinical data to substantiate its efficacy and safety.

This study aims to have a sample size of 110 patients, which will be one of the largest randomized controlled trials in this field. The target is to demonstrate the effect of HSM on nocturia in the elderly, clarify the amount of moxibustion, the distribution pattern of thermosensitive acupoints, the frequency of thermosensitive acupoints and the moxibustion sensation for the treatment of nocturia, and further clarify the regulatory effect of HSM on AVP and ANP, to provide experimental data for clarifying the therapeutic mechanism of HSM on nocturia in the elderly and direction for future basic research. Compared to previous studies (29, 30), our study boasts a shorter treatment duration, which is more readily acceptable to patients. Furthermore, our protocol places emphasis on the long-term maintenance effects of HSM, with reassessments of outcome measures conducted 2 months after treatment. Additionally, we aim to investigate the underlying regulatory mechanisms by analyzing and comparing changes in AVP and ANP levels before and after treatment. To ensure the integrity and scientific validity of the method, every step of the implementation of this clinical study will be strictly carried out, monitored and supervised.

Here are the limitations of our study: Firstly, the inclusion of only a limited number of patients as samples may restrict the representativeness and generalizability of our findings. Secondly, being a single-center study, our research may be influenced by the specific practices, patient demographics, or resource constraints of the institution, thereby limiting the broad applicability of our results. Lastly, with a follow-up period of just 2 months, our study may not adequately capture all significant clinical changes or adverse events associated with a chronic condition such as nocturia.
